# Description of a new Brazilian *Paraportanus* and key to the species of the genus (Insecta, Hemiptera, Cicadellidae, Portanini)

**DOI:** 10.3897/zookeys.242.3528

**Published:** 2012-11-15

**Authors:** Adenomar Neves de Carvalho

**Affiliations:** 1 Instituto de Biodiversidade e Florestas, Universidade Federal do Oeste do Pará, Rua Vera Paz, s/n, Salé, 68035-110, Santarém-PA, Brasil

**Keywords:** Auchenorrhyncha, Xestocephalinae, leafhopper, taxonomy, Amazonian

## Abstract

*Paraportanus longispinus*,, a new leafhopper species from Roraima and Amazonas States, North Brazil, is described and illustrated. The new species can be recognized by the male genital features, especially the distal third of ventral margin of the pygofer with a dentiform short process; plates distinctly longer than pygofer, extending posteriorly beyond pygofer by approximately 1/3 of their length and aedeagus with one pair of spiniform process long crossed and directed ventrally. A checklist and key to males of all known *Paraportanus* species is provided.

## Introduction

The South American genus *Paraportanus* Carvalho & Cavichioli, 2009 is known from ten species (see checklist) from Brazil (Acre, Amazonas, Minas Gerais, Mato Grosso, Maranhão, Pará, Rondônia and Roraima states) and Peru. Among the Portanini, *Paraportanus* species can be recognized by the usually strongly produced male pygofer carrying a pair of strongly pronounced spiniform processes on the posteroventral margin, subgenital plates triangular narrowing to apex and connective Y-shaped with very short stem.

In the present paper, a new *Paraportanus* species from Roraima and Amazonas States (North Brazil) is described and a checklist and key to all known species is provided.

## Material and methods

Techniques for preparation of male genital structures follow Oman (1949). The dissected genital parts are stored in microvials with glycerin and attached below the specimens, as suggested by Young and Beirne (1958). The descriptive terminology adopted herein follows mainly Young (1968, 1977), except for the facial areas of the head (Hamilton 1981).

Label data are given inside quotation marks with a reversed virgule (\) separating lines on the labels and a semicolon separating labels of a specimen.

The specimens studied belong to the Coleção Entomológica Pe. J. S. Moure, Departamento de Zoologia, Universidade Federal do Paraná (DZUP; Curitiba) and Instituto Nacional de Pesquisa da Amazônia (INPA; Manaus).

## Results

### Checklist of *Paraportanus* species

*Paraportanus bicornis* (Carvalho & Cavichioli, 2003)

*Paraportanus bimaculatus* (Carvalho & Cavichioli, 2003)

*Paraportanus cinctus* (Carvalho & Cavichioli, 2003)

*Paraportanus eburatus* (Kamer, 1964)

*Paraportanus elegans* (Kramer, 1961)

*Paraportanus facetus* (Kramer, 1961)

*Paraportanus filamentus* (DeLong, 1980)

*Paraportanus jenniferae* Carvalho & Cavichioli, 2009 (type species),

*Paraportanus longicornis* (Osborn, 1923)

*Paraportanus longispinus* sp. n.

*Paraportanus variatus* (Carvalho & Cavichioli, 2003)

#### Paraportanus longispinus sp. n.

urn:lsid:zoobank.org:act:A852D16B-61DC-460E-9B08-83CFD0A60099

http://species-id.net/wiki/Paraportanus_longispinus

Figs 1 –7

##### Description.

**Figures 1–6.**
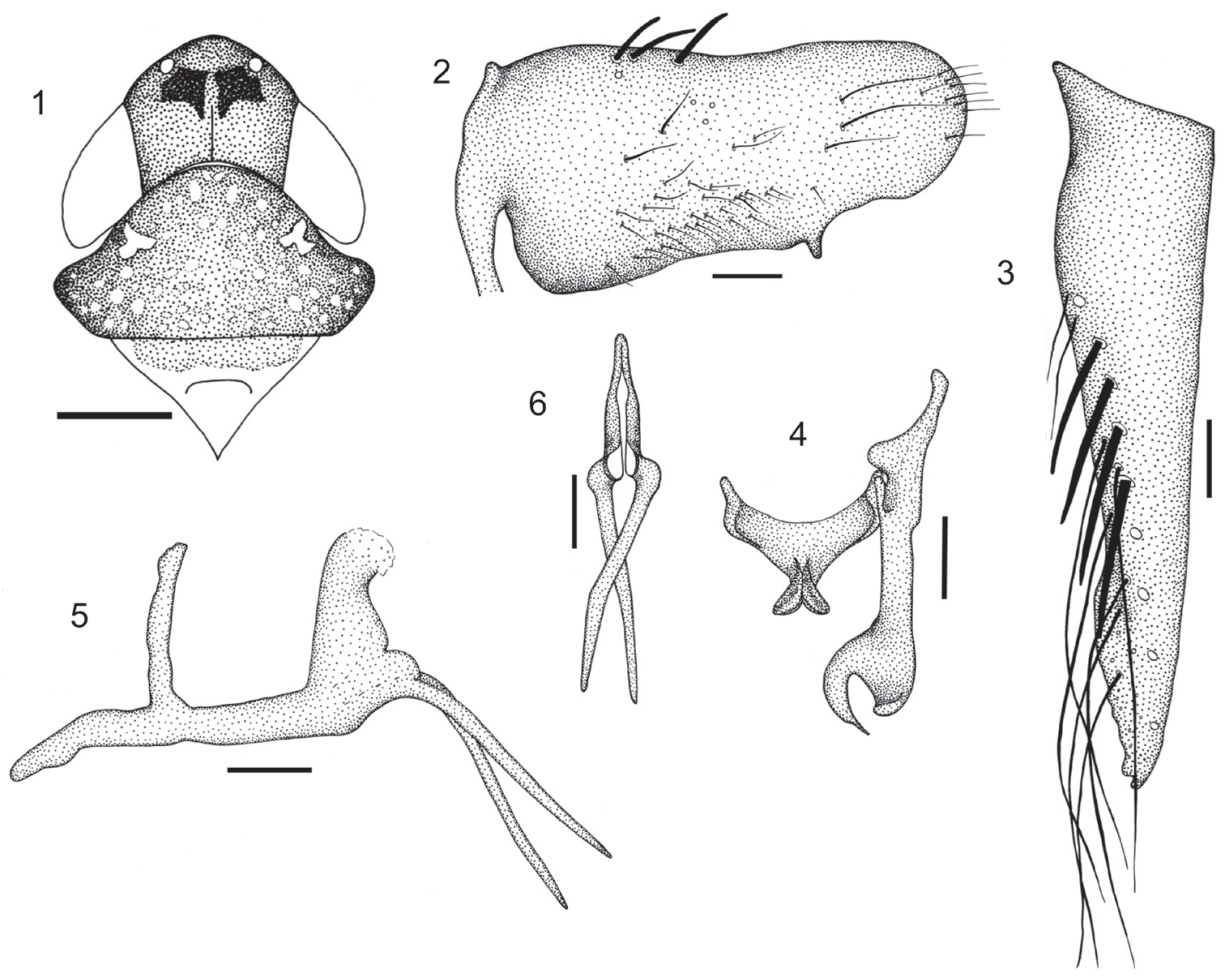
*Paraportanus longispinus*, sp. n., male holotype: **1** head, pronotum and mesonotum, in dorsal view **2** pygofer, in lateral view **3** subgenital plates, in ventral view **4** connective and style, in dorsal view **5** aedeagus, in lateral view **6** apex of aedeagus, in posterior view. Scales 0,15 m

Length 5 mm from apex of head to apex of forewings at rest. Crown (Fig. 1) strongly produced anteriorly; anterior margin rounded in dorsal view; ocelli located on anterior margin, equidistant from the anterior angles of the eyes and coronal suture, the latter half length of crown.

Pronotum (Fig. 1) convex, wider than head; lateral margins subangulate in dorsal view; with dorsopelural carinae; posterior margin straight. Forewings with three closed anteapical cells; median cell as long as the external; third and fourth apical cell subretangular. Others characters as in description generic (Carvalho and Cavichioli 2009).

Color of body light yellow with opaque areas on crown and pronotum. Crown light brown with pair of large black maculae between ocelli (Fig. 1); face with vertical brown stripe on laterofrontal suture, broader adjacent antenna. Forewings light yellow semi-hyaline with white maculae at apex.

Male genitalia with pygofer (Fig. 2) in lateral view, strongly produced posteriorly; posterior margin broadly rounded; distal third of ventral margin with short dentiform process; macrosetae distributed mostly on proximal third of dorsal margin, some smaller setae on ventral margin to apex. Subgenital plates (Fig. 3), elongate, distinctly longer than pygofer, extending posteriorly approximately 1/3 their length, triangular with lateral margin sinuate; ventro laterally with diagonal row of macrosetae over distal two thirds and several long fine setae. Connective (Fig. 4) Y-shaped with arms broadly divergent; stem very short with strong median keel. Styles (Fig. 4) with apical apophysis strongly curved. Aedeagus (Fig. 5) in lateral view, narrow basally; shaft expanded distally, abruptly curved dorsally and laterally compressed; a pair of long spiniform processes subapically on ventral margin (Fig. 6) crossed and directed ventrally; gonopore apical.

**Female genitalia.** The abdominal VII sternite, in ventral view, with anterior margin straight; posterior margin weakly sinuate, with a small rounded tooth medially.

##### Material examined.

Male holotype, “Brasil: Roraima / Rio Uraricoera / Ilha de Maracá 02–13.V.1987; J. A. Rafael, J. E. B. Brasil & L. S. Aquino, *leg*.; DZUP” (DZUP). Paratypes: 4 males, same data as holotype (DZUP). 3 males, “Manaus – AM (2°25'S, 60°O) / Brasil 13.XI.1985 (Biological Dynamics of Forest Fragments Project) / 80 Km de Manaus / Bert Klein, *leg*. / Malaise”; *Ibdem*. 3 males, 01.IV.1985; *Ibdem*. 18.II.1987; *Ibdem*. 1 male, 1 female, 18.IX.1985; *Ibdem*. 1 male, 03.XII.1985; *Ibdem*. 1 male, 17.IX.1985; *Ibdem*. 1 male, 14.I.1985; *Ibdem*. 1 male, 10.XII.1985; *Ibdem*. 1 male, 24.IX.1985; *Ibdem*. 1 male, 19.IX.1985; *Ibdem*. 1 female, 04.XII.1985; *Ibdem*.1 male, 11.XII.1985; *Ibdem*. 1 male, 12.XII.1985; *Ibdem*. 1 male, 24.IX.1985; *Ibdem*. 1 male, 25.IX.1985; *Ibdem*. 1 female, 11.XI.1987; *Ibdem*. 1 male, 25.II.1987; *Ibdem*. 1 female, 07.XI.1985; *Ibdem*. 1 male, 1 female, 18.IX.1985; *Ibdem*. 1 male, 12.XI.1985; *Ibdem*. 3 males, 2 females, 24.IX.1985; *Ibdem*. 1 male, 11.XII.1985; *Ibdem*. 1 male, 1 female, 25.IX.1985; *Ibdem*. 1 female, 18.XI.1985; *Ibdem*. 1 female, 10.XI.1985; *Ibdem*. 1 male, 28.I.1987; *Ibdem*. 1 male, 21.I.1987; *Ibdem*. 1 female, 25.II.1987 (INPA).

**Figure 7.**
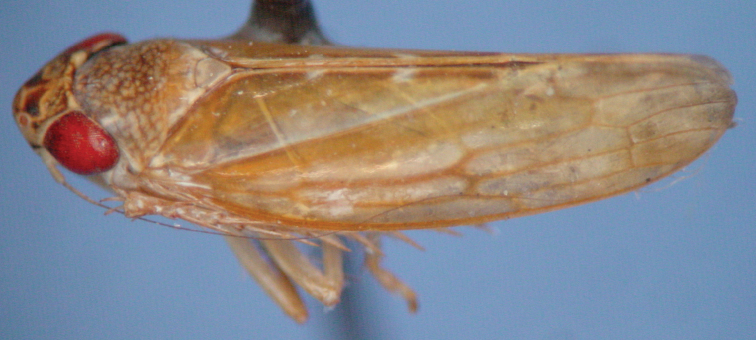
*Paraportanus longispinus* sp. n., male holotype in lateral view.

##### Etymology.

The specific name is named for the long pair of spiniform processes of the aedeagus.

##### Remarks.

*Paraportanus longispinus* can be distinguished from the other known species of the genus by the following combination of features: (1) crown with two maculae between ocelli (Fig. 1); (2) distal third of ventral margin of pygofer with short dentiform process (Fig. 2); (3) aedeagus with a pair of long spiniform process, crossed and directed ventrally (Figs 5, 6). The male genitalia of the new species are similar to those of *Paraportanus facetus* (Kramer) but differ from this species in having the processes of the aedeagus longer and crossed.

### Key to males of *Paraportanus* (modified from Carvalho and Cavichioli 2009 to include *Paraportanus longispinus* sp. n.)

**Table:** 

1	Style with apical portion slightly tapered and twisted	2
–	Styles with apical portion enlarged and bifid (Fig. 4)	4
2	Pygofer with posterior margin truncate with long robust spiniform process	*Paraportanus filamentus*
–	Pygofer with posterior margin angled without such process	3
3	Crown orange with two black subtriangular spots between ocelli	*Paraportanus cinctus*
–	Crown brown with orange spots	*Paraportanus bimaculatus*
4	Aedeagus with pair of lamellar processes	5
–	Aedeagus with pair of spiniform processes	7
5	Connective with lamellar process at confluence of ventral arms; pygofer with spiniform process elongate, curved dorsally, exceeding posterior-dorsal angle	6
–	Connective not as above; pygofer with spiniform process short and stout, curved dorsally, not exceeding posterior-dorsal angle	*Paraportanus eburatus*
6	Crown and pronotum light brown, with minute stramineous spots	*Paraportanus longicornis*
–	Crown orange with pair of dark brown transverse bands behind ocelli; pronotum with stramineous spots and distinct pair of orange spots centrally	*Paraportanus elegans*
7	Pygofer with elongate spiniform process at ventral posterior angle; aedeagus with pre-apical processes short	*Paraportanus jenniferae*
–	Pygofer without elongate spiniform process at posteroventral angle; aedeagus with pre-apical processes short or elongated (Fig. 5)	8
8	Aedeagus with a pair of short processes	9
–	Aedeagus with a pair of long processes (Fig. 5)	10
9	Pygofer, in side view, with the apical margin angulate with short and robust dentiform process pre-apically, directed medially. Scutellum brown, white apically	*Paraportanus variatus*
–	Pygofer, in side view, with the apical margin truncate without process. Scutellum brown with two white spots on lateral margin	*Paraportanus bicornis*
10	Pygofer with posterior margin broadly roundedwith dentiform process (Fig. 2)	*Paraportanus longispinus* sp. n.
–	Pygofer with posterior margin narrowly rounded without dentiform process	*Paraportanus facetus*

## Supplementary Material

XML Treatment for Paraportanus longispinus
